# Characterization of Spontaneous, Transient Adenosine Release in the Caudate-Putamen and Prefrontal Cortex

**DOI:** 10.1371/journal.pone.0087165

**Published:** 2014-01-29

**Authors:** Michael D. Nguyen, Scott T. Lee, Ashley E. Ross, Matthew Ryals, Vishesh I. Choudhry, B. Jill Venton

**Affiliations:** Department of Chemistry, University of Virginia, Charlottesville, Virginia, United States of America; Karolinska Inst, Sweden

## Abstract

Adenosine is a neuroprotective agent that inhibits neuronal activity and modulates neurotransmission. Previous research has shown adenosine gradually accumulates during pathologies such as stroke and regulates neurotransmission on the minute-to-hour time scale. Our lab developed a method using carbon-fiber microelectrodes to directly measure adenosine changes on a sub-second time scale with fast-scan cyclic voltammetry (FSCV). Recently, adenosine release lasting a couple of seconds has been found in murine spinal cord slices. In this study, we characterized spontaneous, transient adenosine release *in vivo*, in the caudate-putamen and prefrontal cortex of anesthetized rats. The average concentration of adenosine release was 0.17±0.01 µM in the caudate and 0.19±0.01 µM in the prefrontal cortex, although the range was large, from 0.04 to 3.2 µM. The average duration of spontaneous adenosine release was 2.9±0.1 seconds and 2.8±0.1 seconds in the caudate and prefrontal cortex, respectively. The concentration and number of transients detected do not change over a four hour period, suggesting spontaneous events are not caused by electrode implantation. The frequency of adenosine transients was higher in the prefrontal cortex than the caudate-putamen and was modulated by A_1_ receptors. The A_1_ antagonist DPCPX (8-cyclopentyl-1,3-dipropylxanthine, 6 mg/kg i.p.) increased the frequency of spontaneous adenosine release, while the A_1_ agonist CPA (N^6^-cyclopentyladenosine, 1 mg/kg i.p.) decreased the frequency. These findings are a paradigm shift for understanding the time course of adenosine signaling, demonstrating that there is a rapid mode of adenosine signaling that could cause transient, local neuromodulation.

## Introduction

Adenosine is an important neuroprotective modulator in the brain that regulates neurotransmission and blood flow. Adenosine increases in the brain during pathological events, such as ischemia [1] and seizures [2], where it can act as a retaliatory metabolite. The increases in adenosine during these pathologies typically last for minutes to hours [3]. For example, the concentration of adenosine doubled one minute following ischemia and was ten-fold larger twenty minutes afterwards [4]. During hypoxia, adenosine activates inhibitory A_1_ adenosine receptors a couple of minutes after onset, which decreases cAMP concentrations, hyperpolarizes neurons, and prevents excitatory firing [5], [6]. While these studies demonstrate adenosine signaling on a longer time scale, there is growing evidence that adenosine also signals on a much shorter time scale.

Using electrophysiological techniques, Dunwiddie’s group explored a rapid modulatory role for adenosine in the brain. During very short electrical stimulations (1–5 pulses at 100 Hz), adenosine regulated glutamate receptor-mediated excitatory postsynaptic potentials (EPSPs) in the hippocampus in an activity dependent manner [7]. The duration of change in EPSPs was only 2 seconds, implying fast adenosine changes were responsible. However, the experiment did not directly measure adenosine concentration on a rapid time scale.

Recently, electrochemical sensors were developed for rapid measurements of changes in local adenosine concentration. Enzyme biosensors can quantitate adenosine with a time resolution of 2 seconds [8]. Using these biosensors, electrically-stimulated adenosine release in the cerebellum was measured that lasted 100 seconds [9]. Our lab developed fast-scan cyclic voltammetry (FSCV) at carbon-fiber microelectrodes [10] to directly detect electrically-stimulated adenosine release in the caudate-putamen that lasts only 10–40 seconds [11]. The concentration of stimulated release was regulated by A_1_ receptors [12]. Using FSCV, the Zylka group recently discovered spontaneous adenosine transients. The adenosine lasted less than 2 seconds in the extracellular space and was a result of extracellular ATP metabolism [13], [14]. These studies establish that adenosine can be released on a more rapid time scale; however, the characteristics and regulation of rapid adenosine signaling *in vivo* are not well understood.

In this study, we measured spontaneous, transient adenosine release *in vivo* for the first time. Spontaneous adenosine release, not evoked by electrical stimulation, was measured in the caudate-putamen and the prefrontal cortex of the anesthetized rat. The duration of direct adenosine release *in vivo* was only about 3 seconds, a time scale that matches the previous electrophysiological study [15]. The concentration of adenosine release was on average 0.18 µM but a large range of concentrations was detected. The frequency of spontaneous adenosine events was one transient every 2–3 minutes and was modulated by A_1_ receptors; however, the events follow a random, not a regular firing pattern. The spontaneous adenosine transients observed *in vivo* were similar in concentration, duration, and frequency to what others have found in murine spinal cord slices [13], suggesting transient adenosine release is common in multiple regions in the nervous system and across different species. This study demonstrates that adenosine is rapidly released and cleared in the brain and may have a rapid neuromodulatory role in addition to the previously characterized function as a long-term modulator.

## Experimental Methods

### Ethics

All animal experiments were carried out under strict accordance with the recommendations in the Guide for the Care and Use of Laboratory Animals of the National Institutes of Health. The protocol was approved by the Institutional Animal Care and Use Committee of the University of Virginia (Protocol Number 3517). All surgery was performed under urethane anesthesia and all efforts were made to minimize suffering.

### Chemicals

All reagents were purchased from Fisher Scientific (Fair Lawn, NJ, USA) unless otherwise stated. Phosphate buffered saline (PBS) was used to calibrate electrodes containing (in mM): 131.25 NaCl, 3.0 KCl, 10.0 NaH_2_PO_4_, 1.2 MgCl_2_, 2.0 Na_2_SO_4_, and 1.2 CaCl_2_ with the pH adjusted to 7.4. Sodium phosphate was purchased from RICCA Chemical Company (Arlington, TX, USA). All aqueous solutions were prepared with deionized water (Milli-Q Biocel; Millipore, Billerica, MA, USA). Adenosine was prepared as a 10 mM stock solution in 0.1 M HClO_4_ and stored in the refrigerator.

DPCPX (6 mg/kg, i.p., 8-cyclopentyl-1,3-dipropylxanthine, Sigma Aldrich) was dissolved in dimethylsulfoxide (DMSO). CPA (N^6^-cyclopentyladenosine) was purchased from Tocris Bioscience (Ellisville, MO, USA), dissolved in saline, and administered at 1 mg/kg. These doses were chosen as large doses that were previously used in the literature [12], [16], [17].

### Electrodes and FSCV

Carbon-fiber microelectrodes were prepared as previously described [18]. Briefly, cylindrical microelectrodes were prepared with 7 µm diameter T-650 carbon-fibers (Cytec Engineering Materials, West Patterson, NJ, USA). Fibers were aspirated into glass capillaries (1.2 mm×0.68 mm; A-M Systems, Inc., Seqium, WA, USA), pulled by a vertical pipette puller (model PE-21; Narishige, Tokyo, Japan) into two microelectrodes, and the extended fiber cut with a scalpel to about 50 µm. The fiber/glass interface was sealed with epoxy [Epon resin 828] (Miller-Stephenson Chemical Co. Inc.; Danbury, CT, USA) and 14% wt m-phenylenediamine heated to 80°C. Excess epoxy was rinsed with acetone and electrodes were dried overnight at room temperature, then cured at 100°C for two hours, and then at 150°C overnight. The Nafion-CNT coated electrodes were prepared as previously described [19]. High pressure carbon monoxide conversion single-walled CNTs (Carbon Nanotechnologies, Houston, TX, USA) were functionalized as described here [20]. The electrodes were dipped in 0.05 mg/mL CNTs suspended 5% wt Nafion in methanol (Ion Power, New Castle, DE, USA) for 5 minutes, air dried for 10 seconds, placed in the oven for 10 minutes at 70°C, and stored at room temperature overnight. Electrical connections were created by backfilling the electrodes with 1 M KCl. Bare electrodes were soaked in 2-propanol for at least 10 minutes before use.

Fast-scan cyclic voltammetry (FSCV) was used to monitor adenosine with sub-second temporal resolution [10]. Waveform generation and data collection was computer controlled by Tar Heel CV (gift of Mark Wightman, UNC at Chapel Hill) [21]. A Dagan ChemClamp potentiostat (Dagan Corporation; Minneapolis, MN, USA) was used to collect data. Electrical stimulations were applied with a BSI-950 Bi-Phasic Stimulus Isolator (Dagan).

Electrodes were continuously held at −0.40 V and scanned to 1.45 V and back every 100 milliseconds against a Ag/AgCl reference electrode, at a rate of 400 V/s. All data was background subtracted to remove any non-Faradic currents by averaging 10 CVs from no more than 20 seconds before the analysis point. Electrodes were calibrated using flow injection analysis [22] with 1.0 µM adenosine in PBS made fresh daily from the 10 mM stock solution. Pre- and post-calibrations were performed and no significant difference in adenosine oxidation current was found after electrode implantation (data not shown).

### Animals and Surgery

Male Sprague-Dawley Rats (250–350 g; Charles-River, Wilmington, MA, USA) were housed in a vivarium with 12-h light/dark cycles and provided food and water *ad libitum*. The rats were anesthetized with 50% wt urethane (Sigma Aldrich) solution in saline (Baxter; Deerfield, IL, USA) (1.5 g/kg, i.p). The surgical site was shaved and 0.25 mL of bupivicaine (Sensorcaine® MPF, APP Pharmaceuticals, LLC; Schaumburg, IL, USA) was administered subcutaneously for local analgesia. After exposing the skull, holes were drilled for placement of electrodes using a stereotaxic drill [23]. Adenosine transients were measured in either the caudate or the prefrontal cortex in each rat. The coordinates for the caudate-putamen are (in mm from bregma): anterior-posterior (AP): +1.2, mediolateral (ML): +2.0, and dorsoventral (DV): −4.5. Coordinates for the prefrontal cortex are: AP: +2.7, ML: +0.8, and DV: −3.0. The Ag/AgCl reference electrode was inserted on the contralateral side of the brain. The rat’s body temperature was maintained at 37°C using a heating pad with a thermistor probe (FHC, Bowdoin, ME, USA).

### Data Collection and Analysis

Electrodes were implanted into the tissue and allowed to equilibrate with the waveform being applied for at least 30 minutes before any data collection. After equilibration, data was continuously collected and if no transients were found after 30 minutes or if the electrode baseline was unstable, a new electrode was inserted until transients were observed. Up to three electrodes per animal were inserted to search for transient adenosine release. Any animals with fewer than four transients in the hour long pre-drug time period were excluded. The overall success rate for finding transients was 80% in both the caudate-putamen and in the prefrontal cortex. After electrode placement was deemed optimal, one hour of pre-drug data was collected and then drug was injected. Nafion-CNT coated electrodes were placed *in vivo* and data collected for one hour.

### Principal Component Analysis

All adenosine transients were qualitatively and quantitatively analyzed using High Definition Cyclic Voltammetry (HDCV) Analysis software (from Mark Wightman, UNC at Chapel Hill). A training set was compiled for each rat of the five largest, most definitive adenosine transients with a clear secondary peak. The largest transients were chosen because they were easily identifiable, whereas smaller transients created poor principal component correlations. Principal components were extracted from the training set and all data was analyzed using principal component regression [24]. This produced an adenosine concentration vs. time trace that was used to identify and determine the amount and duration of each transient. Every training set has residuals which account for currents from unknown signals, such as noise [25]. The sum of squares of the residuals for each variable, or the Q score, was calculated and any signal above Q failed and was not counted as an adenosine transient. With a limit of quantitation at ten times the noise for the secondary peak, the smallest transients that could be quantified were 40 nM. Any transient signal without a secondary peak or any sustained signal greater than 10 seconds, where the background could not be accurately subtracted out, was not counted.

### Statistics

Statistics performed using GraphPad PRISM 6 (GraphPad Software Inc., San Diego, CA, USA), MatLAB® (The MathWorks, Inc., Natick, MA, USA) and OriginPro 7.5 (OriginLab Corporation, Northampton, MA, USA). Data presented as mean ± SEM with *n* number of animals. A Kolmogorov-Smirnov (KS) test was used to determine underlying distributions between inter-event times (time between consecutive transients). All data was considered significant at the 95% confidence level.

## Results

### Adenosine Detection using FSCV

**Figure 1:**
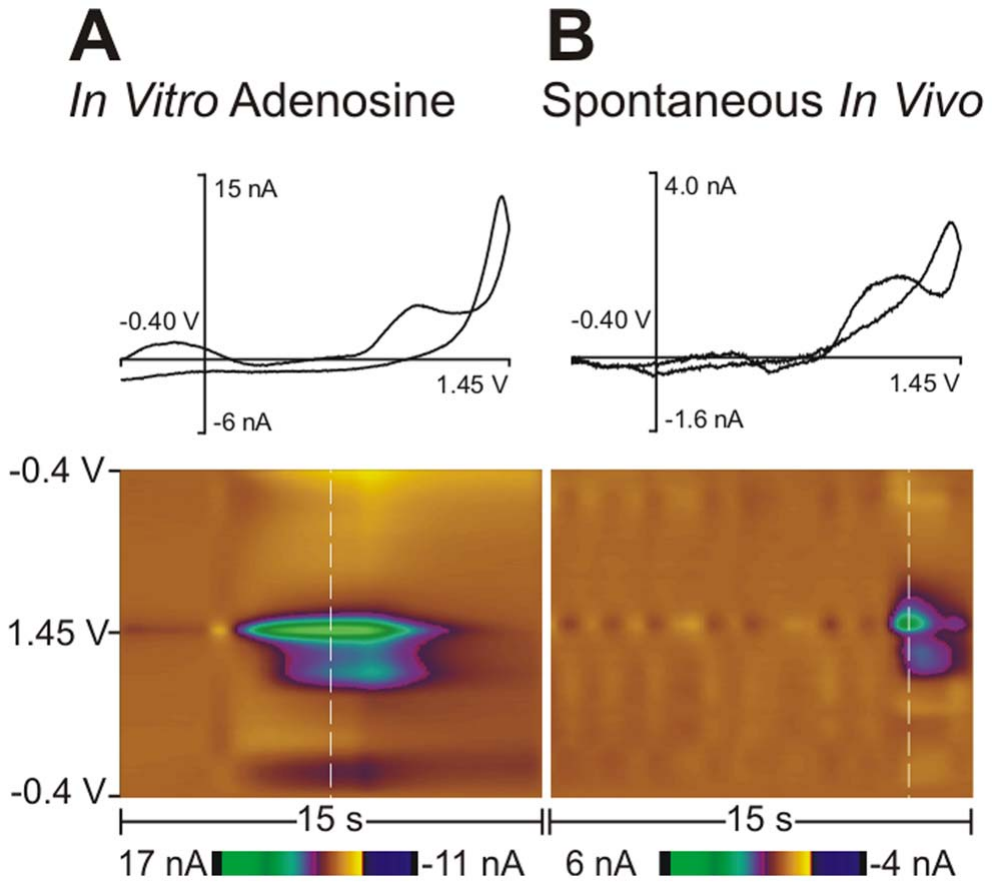
Detection of adenosine *in vitro* and *in vivo* using fast-scan cyclic voltammetry at the same carbon-fiber microelectrode. (A) *In vitro* calibration of adenosine. A 3-D color plot (bottom) depicts time on the *x*-axis, potential on the *y*-axis, and current in false color. The primary oxidation occurs at 1.4 V (large green oval in center of color plot) and the secondary oxidation occurs at 1.0 V (green/purple oval below center oval). The dashed white line on the color plot denotes where the background subtracted cyclic voltammogram (CV, top) is obtained. (B) *In vivo* spontaneous, transient adenosine event. The 3-D color plot and CV shows primary and secondary oxidation peaks that match the *in vitro* calibration.

Spontaneous adenosine transients were monitored using FSCV, which has sub-second temporal resolution, allowing real time measurements of adenosine changes in the brain. No information about basal levels is obtained with FSCV because all data are background subtracted. The carbon-fiber microelectrode was scanned from −0.40 V to 1.45 V and back at 10 Hz. Adenosine undergoes two sequential, two electron oxidations [26] that produce two peaks in the background-subtracted cyclic voltammogram (CV) [10] (Fig. 1A top). Both oxidation peaks are evident in the color plot of an electrode calibration with 1.0 µM adenosine (Fig. 1A). The primary oxidation occurs at 1.4 V and the secondary oxidation at 1.0 V is slightly delayed in time from the main oxidation peak because the secondary product forms after the primary product is produced.

Spontaneous adenosine release was identified by its two oxidation peaks in the CVs and color plots, which help distinguish adenosine from other compounds with similar oxidation potentials including hydrogen peroxide [27], histamine [28], and hypoxanthine [29]. In addition, the slight delay in the formation of the second peak helps identify the analyte as adenosine [30]. While adenosine and ATP have similar cyclic voltammograms, carbon-fiber electrodes are more sensitive for adenosine than ATP [10] and ATP breaks down to adenosine in the extracellular space within 200 milliseconds [31]. As an additional test to confirm adenosine was detected and not ATP, we used Nafion-CNT coated electrodes, which are three times more sensitive for adenosine than ATP compared to bare carbon-fiber microelectrodes. Spontaneous adenosine transients were found with Nafion-CNT electrodes implanted in the caudate-putamen with similar concentrations (Nafion-CNT average = 0.13±0.01 µM, *n* = 3, unpaired *t*-test, *p* = 0.1751). The duration of the Nafion-CNT transients were longer than those detected with bare electrodes (average = 3.8±0.2 seconds, *n* = 3, unpaired *t*-test, *p* = 0.0002). Nafion is known to slow the temporal response of electrodes so longer durations are due to a slower electrode response [32]. The similar magnitude of transient release suggests that the signal is from spontaneous adenosine release and not ATP release. Due to the labor intensive process of making Nafion-coated electrodes and the loss of temporal resolution, bare electrodes were used in all the other experiments.

Representative color plots and matching CVs from the same electrode are shown for an *in vitro* calibration of adenosine (Fig. 1A) and a spontaneous adenosine transient (Fig. 1B) in the caudate-putamen. The cyclic voltammograms of *in vitro* adenosine and spontaneous adenosine release *in vivo* show a clear primary oxidation peak at 1.4 V and a definitive secondary peak at 1.0 V. The CV for the calibration was similar to the *in vivo* transient and there was an R^2^ correlation value of 0.81 between the two CVs. The color plots illustrate that both have a minor delay in the secondary peak formation. Stimulated adenosine release has been previously characterized in our laboratory [11], but the secondary peak was not consistently observed due to the overall small signals and other chemical changes. The CVs for stimulated adenosine can be convoluted with residual dopamine, pH, and oxygen changes [33]. Thus, stimulated adenosine release was not a good comparison for identifying spontaneous adenosine release.

### Automated Identification of Spontaneous Adenosine Transients

**Figure 2:**
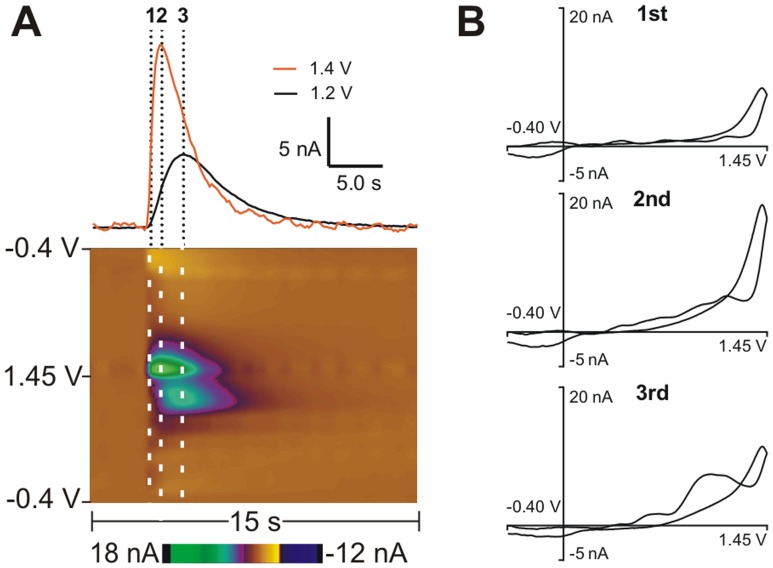
Detection of spontaneous, transient adenosine release *in vivo*. (A) *In vivo* spontaneous, transient adenosine release. The current vs. time plot has two traces, an orange line at 1.4 V for the primary oxidation and a black line at 1.2 V for the secondary oxidation. The dashed lines on the color plot and current vs. time plots indicate where CVs were taken. (B) Cyclic voltammograms of adenosine over time. The top (1^st^) is when adenosine first appears, the middle CV (2^nd^) is half a second later when the primary peak is at a maximum and the bottom (3^rd^) is half a second later when the secondary peak is at its maximum. The ratio of the primary and secondary oxidation peaks can change over time.

An automated system was needed to identify transient adenosine release events without bias. Principal components analysis (PCA) [34] has been previously used to identify spontaneous dopamine transients [35]; therefore we adapted PCA for use with adenosine. Unlike dopamine release [36], the CV of adenosine is not constant over time, due to the secondary product having a delayed increase in current. Figure 2A shows a color plot of a spontaneous adenosine transient with a current vs. time trace (top) through the primary (orange) and secondary (black) oxidation peaks overlaid. The maximum signal at the second peak is 1.0 second after the first peak, demonstrating the lag time between the primary and secondary oxidation peaks. Cyclic voltammograms (Fig. 2B) were taken at sequential times and show that the CV of adenosine release changes over time. The first CV has a primary oxidation peak and no secondary peak. Half a second later, the primary peak is more prominent while the secondary peak has grown, and in the third CV, the secondary and primary peak are almost equal in magnitude. The CVs for the training set of spontaneous adenosine release were taken at the apex of the primary oxidation peak, which gives the most accurate calibration of adenosine. Although the residuals were higher for adenosine than for dopamine because of the variable CVs, adenosine transients were still easily determined at a 95% confidence level.

A training set was created for each individual rat from five of the largest, easily identifiable spontaneous adenosine transients, each containing a characteristic secondary peak. From the training set, the eigenvalues were calculated for CVs of varying concentrations of adenosine. The highest eigenvalues correspond to the principal components with the highest variance, and thus the best correlation to the data [37]. A residual Q-score from the training set was used to reject any data that did not significantly match the principal components. After removing the residuals, the concentration and duration of spontaneous adenosine release was determined from a concentration vs. time plot. The limit of quantitation for our electrodes was 40 nM, so any transients with concentrations below this value were excluded. Below 40 nM, the secondary peak is difficult to identify and is obscured by noise. Thus, for a transient to be considered adenosine, it had to have a secondary peak, be below the residual Q-score (corresponding to 95% confidence level), and be above 40 nM, the quantitation limit.

### Concentration of Spontaneous Adenosine Transients

**Figure 3:**
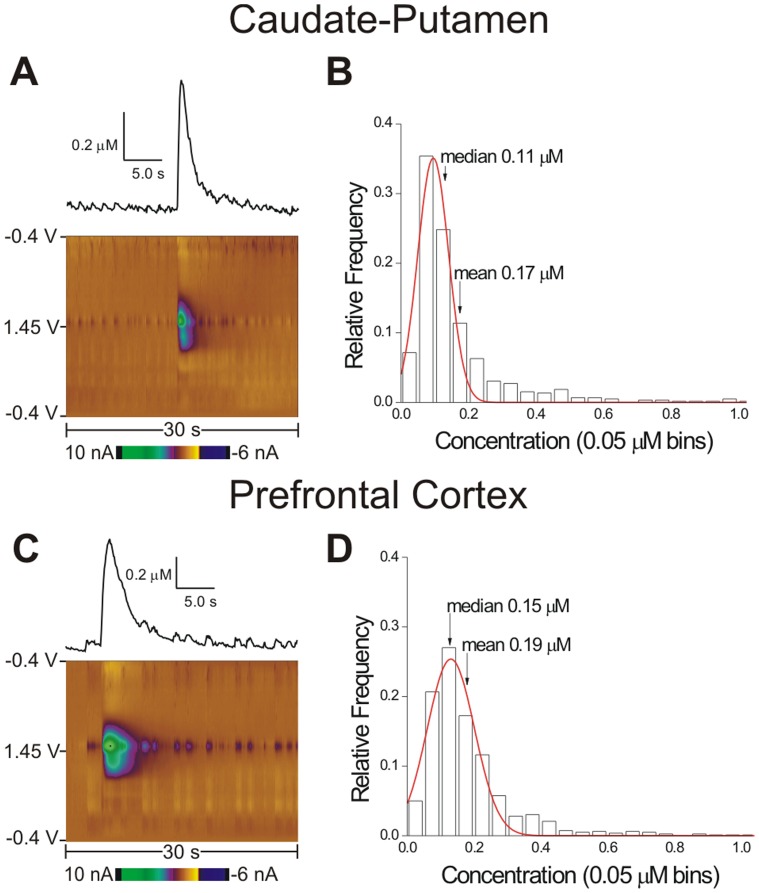
Spontaneous adenosine transient concentration. (A) A color plot of a spontaneous adenosine transient in the caudate putamen with a corresponding concentration vs. time plot above. (B) Caudate-putamen concentration histogram. The *y*-axis is relative frequency. All concentrations from the first hour of data collection in the caudate putamen were placed into 0.05 µM bins (*x*-axis) and fit with a Gaussian distribution (red line). The Gaussian fit equation is 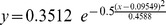 (R^2^ = 0.9327, *n* = 30 rats). The mean and median are marked. The majority of transients are in the 100–200 nM range. (C) An example color plot and concentration vs. time plot in the prefrontal cortex. (D) The histogram of concentrations in the prefrontal cortex fit a Gaussian distribution with the equation: 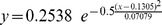 (R^2^ = 0.9505, *n* = 29 rats).

Transient adenosine release was examined in the caudate-putamen and prefrontal cortex. The color plots and concentration vs. time profiles of example adenosine transients show the magnitude of concentration released, as well as the duration of adenosine in the extracellular space in the caudate-putamen (Fig. 3A) and the prefrontal cortex (Fig. 3C). The average adenosine concentration was 0.17±0.01 µM in the caudate-putamen and 0.19±0.01 µM in the prefrontal cortex, which is significantly different (*n* = 30 and 29 rats, *t*-test, *p* = 0.0238). Table 1 gives the average, SEM, and range for the concentration in each brain region. The range of recorded adenosine transients was large, spanning almost two orders of magnitude. While the majority of adenosine release events were in the hundreds of nM range, 1% of transients were greater than 1 µM, which demonstrates that large amounts of adenosine can be spontaneously released. To further investigate spontaneous adenosine release, concentrations were placed in 0.05 µM bins and histograms examined for both brain regions. The histograms have normal distributions and are overlaid with Gaussian fits with positive skews, which is expected when non-negative values are excluded. In the caudate-putamen (Fig. 3B) over half of the values fall between 0.05 and 0.15 µM, although the average value was 0.17 µM, a reflection of the positive skew. Similarly, in the prefrontal cortex (Fig. 3D), a little less than half of the measurements fall between 0.05 and 0.15 µM concentrations and the average is 0.19 µM. Both graphs show that although the bulk of transient adenosine events are in the 0.10 µM range, about 4% of release is 0.50 µM and higher.

**Table 1:** Averages for spontaneous adenosine release.

Caudate-Putamen			
	*Avg.*	*SEM*	*Range*
Concentration (µM)	0.17	0.01	0.04–2.5
Duration (s)	2.9	0.1	0.8–9.9
Inter-event Time (s)	*156	8	1.4–1405
**Prefrontal Cortex**			
	***Avg.***	***SEM***	***Range***
Concentration (µM)	0.19	0.01	0.04–3.2
Duration (s)	2.8	0.1	0.3–10
Inter-event Time (s)	*108	6	0.9–2069

**Legend:** Data from the caudate-putamen and prefrontal cortex. Concentration was significantly different between the two regions (*n* = 588 transients in 30 rats and 804 transients in 29 rats, *t*-test, *p* = 0.0238, respectively). The duration of transient adenosine was not different between the caudate and prefrontal cortex (*n* = 30 and 29 rats, *t*-test, *p* = 0.0826). * Inter-event times, or the time between consecutive transients have significantly different underlying distributions between the two regions (*n* = 30 and 29 rats, KS test, *p* = 0.0338).

### Duration of Transient Adenosine Release

**Figure 4:**
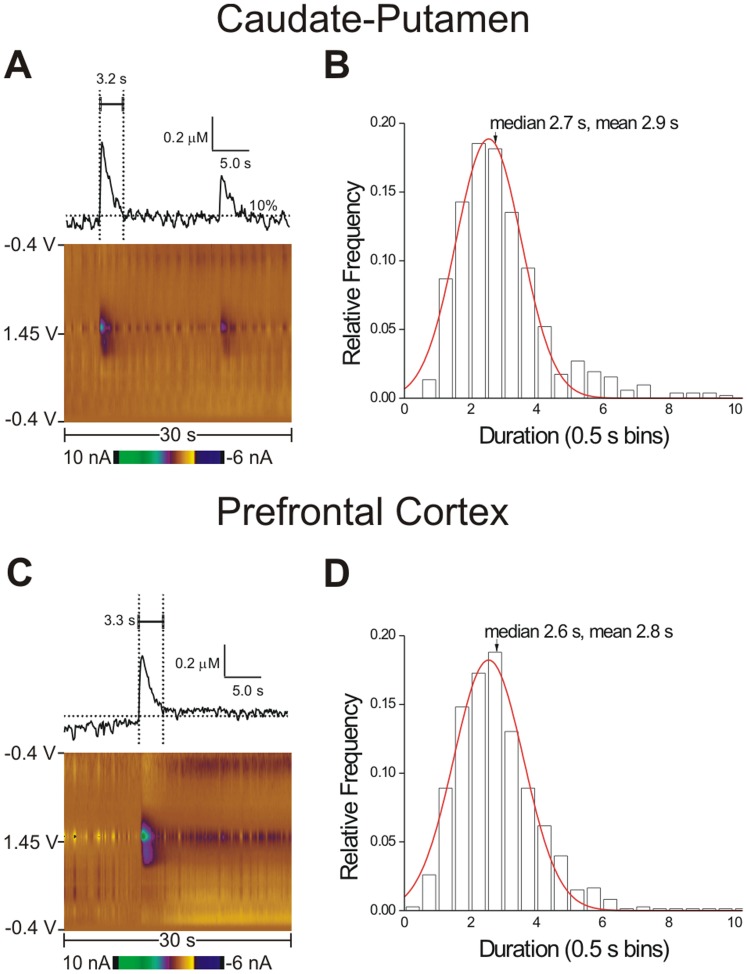
Spontaneous adenosine duration. (A) A color plot and concentration vs. time plot of adenosine transients in the caudate-putamen. The horizontal dashed line is 10% of the concentration of the first peak and the vertical lines are when the baseline value crosses this value, showing the duration. (B) Caudate-putamen duration histogram. The *y*-axis is relative frequency and the *x*-axis shows 0.5 second bins. The Gaussian distribution equation is 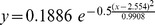 (red line, R^2^ = 0.9712, *n* = 30 rats). (C) Color plot and concentration vs. time plot of an example spontaneous adenosine transient in the prefrontal cortex. The duration is marked with vertical lines. (D) Adenosine duration histogram for the prefrontal cortex is plotted with a Gaussian distribution equation 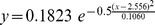 (red line, R^2^ = 0.9765, *n* = 29 rats).

The duration of adenosine release provides information about how long adenosine is available for signaling in the extracellular space. Color plots of typical adenosine transients with corresponding concentration vs. time plots from PCA (above) show adenosine is rapidly cleared in the caudate-putamen (Fig. 4A) and the prefrontal cortex (Fig. 4C). From the top plots the duration was calculated as the amount of time adenosine was over 10% (horizontal dashed line) of the peak concentration for each transient to eliminate any effects from noise in the baseline. The average duration of an adenosine transient was 2.9±0.1 seconds in the caudate-putamen and 2.8±0.1 seconds in the prefrontal cortex, which is not significantly different (*n* = 30 and 29 rats, *t*-test, *p* = 0.0826). The duration ranged from less than a second to ten seconds (Table 1). Histograms of the duration of spontaneous adenosine transients were plotted using 0.5 second bins for the caudate-putamen (Fig. 4B) and the prefrontal cortex (Fig. 4D). The plots are overlaid with a Gaussian distribution fit with positive skews. The majority of transients lasted only 2–4 seconds, but outliers were present in both brain regions. Thus, adenosine is only available for signaling for a few seconds.

### Frequency of Adenosine Transients

**Figure 5:**
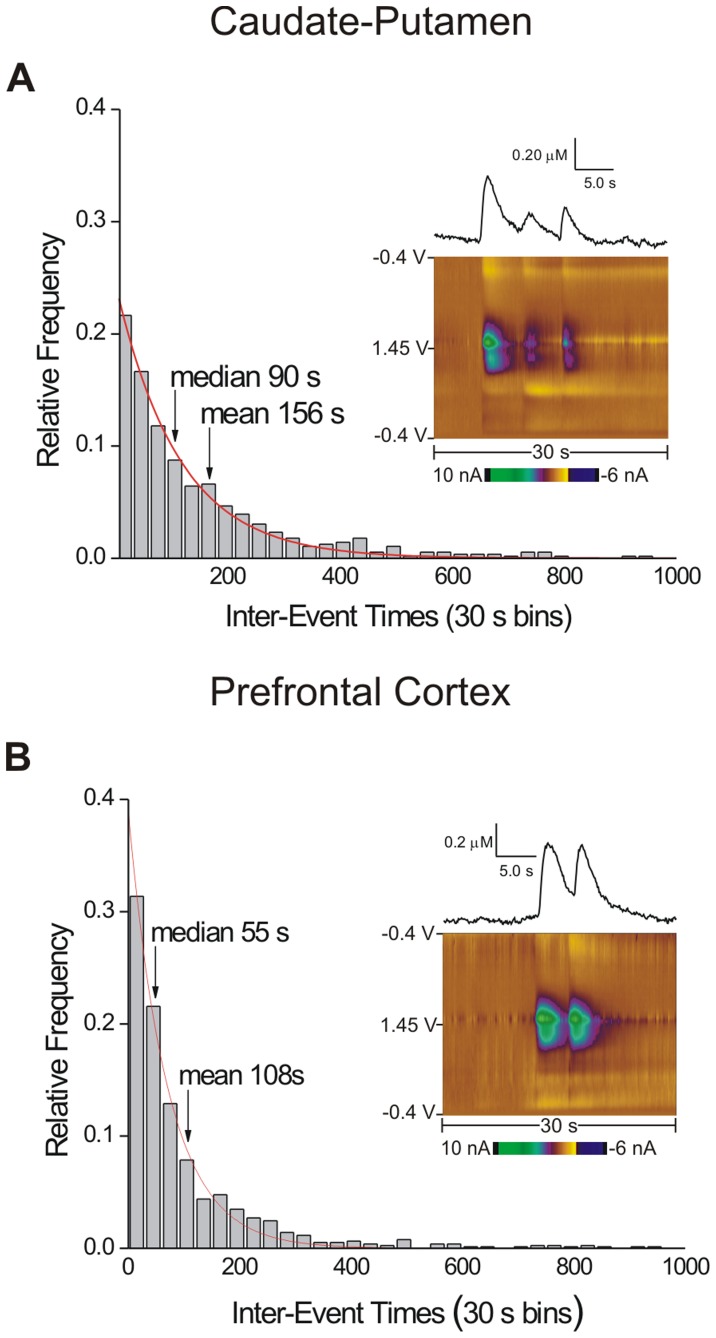
Histograms of inter-event times. (A) Inter-event histograms in the caudate-putamen. The time between consecutive transients (termed the inter-event time) was calculated and plotted for the first hour of data collection. The *x*-axis shows 30 second time bins and the *y*-axis is relative frequency of inter-event times. Median, mean and exponential fit (y = 0.242 *e* ^−0.00891x^ (R^2^ = 0.9926)) are plotted on the histogram. The inset plots show an example of three consecutive transients. (B) Inter-event histograms for the prefrontal cortex. The exponential fit is *y* = 0.388 *e* ^−0.0141x^ (R^2^ = 0.9933). The inset color plot shows an example of two transients that occurred close together. The underlying distribution of inter-event times was significantly different between the caudate-putamen and prefrontal cortex (*n* = 30 and 29 animals, 588 and 804 inter-event times respectively, Kolmogorov-Smirnov test, *p* = 0.0338). The time between transients is shorter in the prefrontal cortex.

On average, spontaneous, transient adenosine release occurs once every several minutes (Table 1). The inter-event times, or the time between consecutive transients, were calculated to examine if the adenosine transients were regularly spaced. Histograms of inter-event times are plotted for the caudate (Fig. 5A) and prefrontal cortex (Fig. 5B), with the median and mean values marked. Relative frequency is plotted on the *y*-axis and 30 second time bins are on the *x*-axis. The distribution was not Gaussian and shows that adenosine transients occur closer together than expected from mean inter-event values. The average time between transients for the caudate putamen was 156 seconds, however, the majority of transients occurred within 2 minutes of each other. Similarly, in the prefrontal cortex, the mean inter-event time was 108 seconds, but a majority of transients happened less than a minute apart. Thus, adenosine release occurs randomly and is not a result of pacemaker firing. Color plots show that occasionally spontaneous release events occur within a couple of seconds of each other (Fig. 5A inset) and the amount of adenosine release does not always decrease with the sequential release (Fig. 5B inset).

The underlying distributions between the two brain regions were compared and the patterns are significantly different (*n* = 30 and 29 rats, Kolmogorov-Smirnov test, *p* = 0.0338). Thus, the time between adenosine transient events was shorter in the prefrontal cortex than in the caudate-putamen. An exponential decay was fit to the data and plotted on the histogram. The exponential fit in the caudate-putamen has a rate constant of 0.00891 s^−1^ or a firing rate of every 112 seconds. The rate constant in the prefrontal cortex is 0.0141 s^−1^ or a firing rate of every 71 seconds.

### Adenosine Transients Continue Over Time

**Figure 6:**
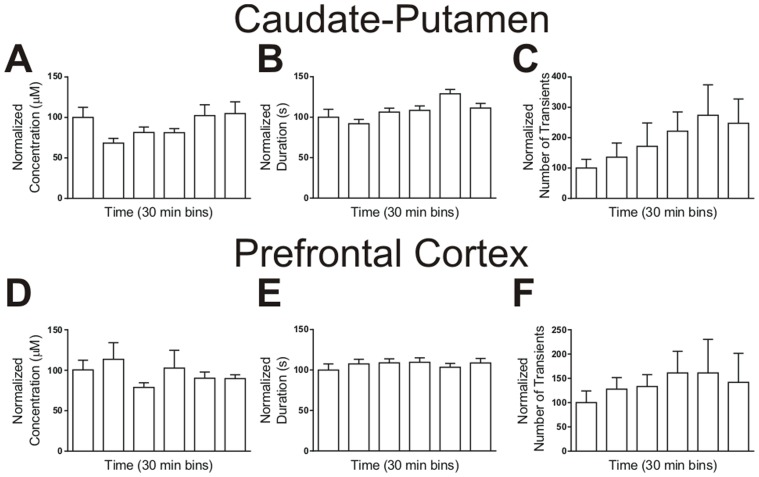
Spontaneous transient adenosine over time. All *x*-axes are time in 30 minute bins and all *y*-axes were normalized to the first bin. (A) Normalized concentrations of adenosine transients in the caudate putamen. There was no significant effect of time on the concentration (*n* = 6 animals, one-way ANOVA, *p* = 0.2013). (B) Duration of adenosine release from the caudate-putamen. There was a main effect of time on durations (*n* = 6 animals, one-way ANOVA, *p* = 0.0005). (C) Number of transients in the caudate-putamen. There was no significant effect of time on the number of transients (*n* = 6 animals, one-way ANOVA, *p* = 0.4782). (D) Concentrations in the prefrontal cortex. There was no significant effect of time on the concentration (*n* = 6 animals, one-way ANOVA, *p* = 0.6268). (E) Durations of transient adenosine in the prefrontal cortex. There was no significant effect of time on the duration (*n* = 6 animals, one-way ANOVA, *p* = 0.8449). (F) Number of transients from the prefrontal cortex. There was no significant effect of time on the number of transients (*n* = 6 animals, one-way ANOVA, *p* = 0.9299).

To examine adenosine transients over time, data were collected continuously over three hours. Figures 6A and 6D display the average concentration of adenosine transients in 30 minute bins in the caudate and prefrontal cortex, respectively. The average concentration in both brain regions was around 0.13 µM and did not significantly differ with time (one-way ANOVA, caudate: *n* = 6, *p* = 0.2013; prefrontal cortex: *n* = 6, *p* = 0.6268). The duration of adenosine release was also binned into 30 minute epochs for the caudate-putamen (Fig. 6B) and the prefrontal cortex (Fig. 6E). There was a significant effect of time on event duration between the 30 minute bins in the caudate-putamen (*n* = 6, one-way ANOVA, *p* = 0.0005) but not the prefrontal cortex (*n* = 6, one-way ANOVA, *p* = 0.8449). The duration of the fifth bin was significantly longer than the durations of the first and second bins in the caudate-putamen (Bonferroni post-test, one-way ANOVA *p*>0.05); however, since the sixth bin was lower, we conclude there is no trend of increasing duration over time. Figures 6C and 6F show the average number of transient release events normalized in 30 minute time bins. The large error bars were due to the large variability in the number of transients from animal to animal. There was no significant effect of time on the average number of transients, indicating time after implantation had no effect in the caudate-putamen (*n* = 6, one-way ANOVA, *p* = 0.4782) or the prefrontal cortex (*n* = 6, one-way ANOVA, *p* = 0.9299). These results indicate that the number, concentration, or duration of adenosine transients does not change for at least three hours following electrode implantation.

### A_1_ Receptor Modulation

**Figure 7:**
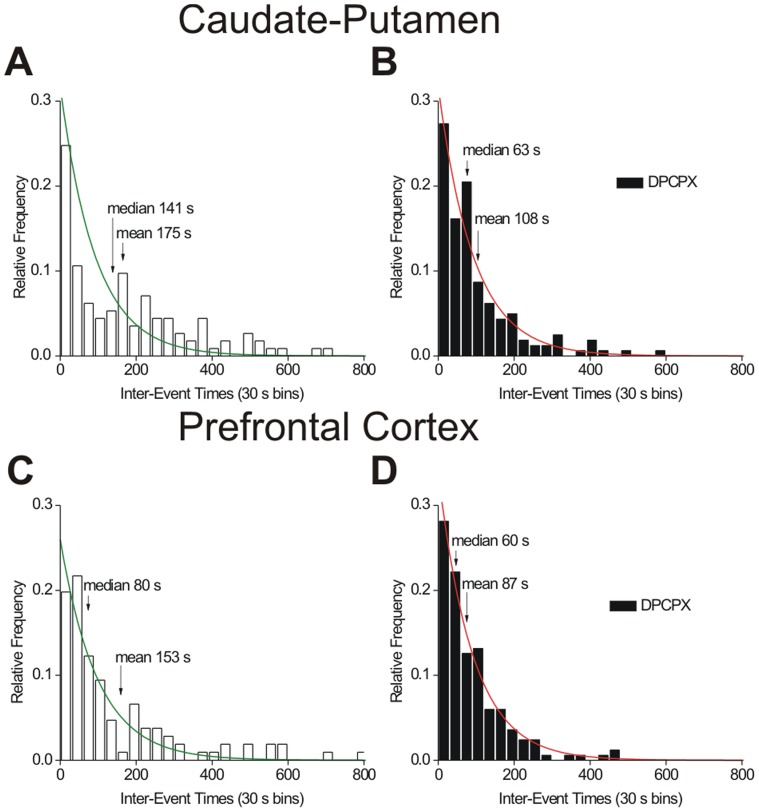
Effect of the A_1_ antagonist, DPCPX (6 mg/kg, i.p.), on adenosine transients. (A) Inter-event time histograms of first hour of pre-drug in caudate. Median, mean and exponential fit (green line) (y = 0.295 *e* ^−0.0186^ (R^2^ = 0.7889)) are plotted on the frequency distribution. (B) Inter-event time histogram for transients in the first hour post-DPCPX in caudate. The exponential fit (red line) is y = 0.318 *e* ^−0.0101x^ (R^2^ = 0.9490). In the caudate, there was a significant difference between the underlying distributions before and after DPCPX (*n* = 6 animals, KS-test, *p*<0.0001). (C) Inter-event histograms pre- and (D) post-DPCPX in the prefrontal cortex. The exponential fit equations are y = 0.260 *e* ^−0.0101x^ (R^2^ = 0.9124) pre-drug and y = 0.340 *e* ^−0.0111x^ (R^2^ = 0.9851) after DPCPX. In the prefrontal cortex, there was a significant difference between underlying distributions pre- and post-DPCPX (*n* = 5 animals, KS-test, *p* = 0.0287).

Previous studies found A_1_ receptors modulate stimulated adenosine release [12], so we tested the effects of A_1_ receptor drugs on spontaneous adenosine release. For all drug experiments, after the electrode was equilibrated, one hour of baseline data was collected and then the drug administered. DPCPX, an A_1_ receptor antagonist, was administered at 6 mg/kg, i.p. The CVs of adenosine transients observed after DPCPX were similar to those observed before drug, indicating that DPCPX did not interfere with the detection of adenosine using FSCV. The concentration and duration of adenosine release were compared before and after DPCPX using paired *t*-tests to examine release in the same animal. DPCPX did not significantly affect the concentration of release in the caudate (*n* = 6, paired *t*-test, *p* = 0.1186) or prefrontal cortex (*n* = 5, paired *t*-test, *p* = 0.8547). However, in the caudate-putamen, DPCPX significantly increased the duration from 2.6 to 3.0 seconds (*n* = 6, paired *t*-test, *p* = 0.0092). The duration of adenosine release in the prefrontal cortex increased from 2.4 to 2.9 seconds after DPCPX, but the increase was not significant (*n* = 5, paired *t*-test, *p* = 0.1300). Following DPCPX administration, there was a decrease in the mean and median inter-event times and a significant difference in the underlying frequency distribution in the caudate-putamen (*n* = 6 rats, KS test, *p*>0.0001) (Fig. 7A and 7B) and the prefrontal cortex (*n* = 5 rats, KS test, *p* = 0.0286) (Fig. 7C and 7D). The time between events decreased after DPCPX administration from a median of 141 seconds pre-drug to 63 seconds in the caudate and from 80 seconds to 60 seconds in the prefrontal cortex. Therefore, blocking A_1_ receptors with DPCPX in both brain regions increases the frequency of spontaneous, transient adenosine release.

**Figure 8:**
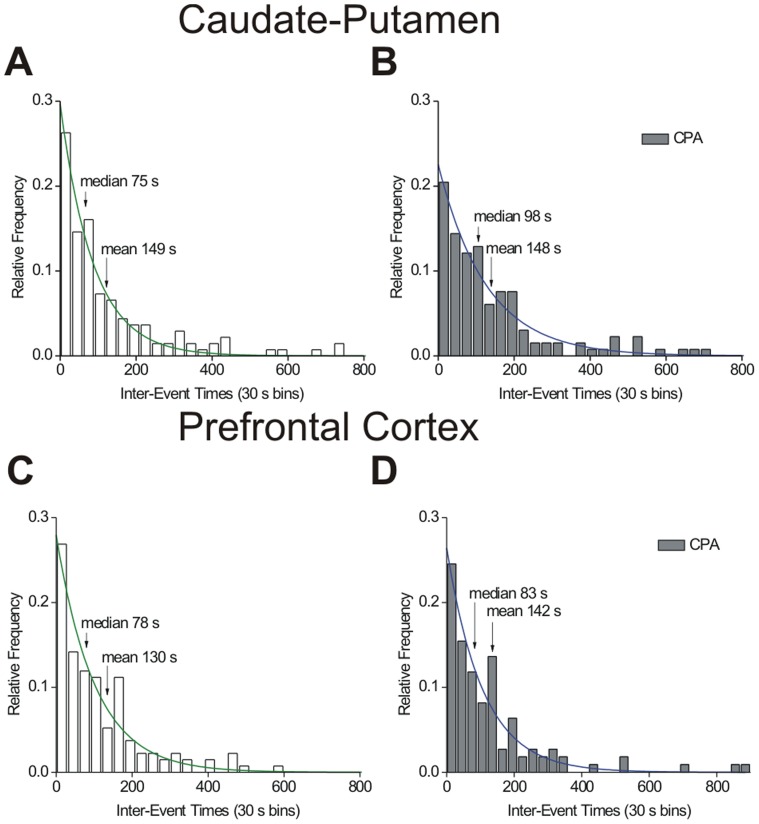
Effect of the A_1_ agonist, CPA (1 mg/kg, i.p.), on adenosine transients. (A) Inter-event time histograms for the caudate. Median, mean and exponential fit (green line) (y = 0.298 *e* ^−0.0115x^ (R^2^ = 0.9636)) are plotted on the frequency distribution. (B) Inter-event time histograms for the first hour after CPA in caudate. The exponential fit (blue line) is y = 0.226 *e* ^−0.00787x^ (R^2^ = 0.9528). There was a significant difference between the underlying distributions before and after CPA (*n* = 6 animals, KS-test, *p* = 0.0308). (C) Inter-event histograms pre- and (D) post-CPA in the prefrontal cortex. Exponential equations are y = 0.280 *e* ^−0.00983x^ (R^2^ = 0.9430) pre-drug and y = 0.264 *e* ^−0.00938x^ (R^2^ = 0.9337) after CPA. In the prefrontal cortex, there was no significant difference in the underlying distributions before and after CPA (*n* = 6 animals, KS-test, *p* = 0.9299).

The A_1_ agonist, N^6^-cyclopentyladenosine (CPA), was administered to determine if activation of A_1_ receptors affected spontaneous adenosine transients. There was no significant change in adenosine concentration in either the caudate or the prefrontal cortex following administration of CPA (paired *t*-test, *n* = 6, *p* = 0.9818; *n* = 6, *p* = 0.4607, respectively). However, CPA did increase the duration of adenosine transients in the caudate from 2.7 to 3.4 seconds (*n* = 6, paired *t*-test, *p* = 0.0195) and from 3.2 to 3.8 seconds in the prefrontal cortex (*n* = 6, paired *t*-test, *p* = 0.0259). The drugs may interfere with uptake or metabolism, thus increasing duration. In the caudate-putamen, the median and mean inter-event times increased after CPA (Fig. 8A and 8B) and the underlying distributions were significantly different than pre-drug (*n* = 6 rats, KS test, *p* = 0.0308). The time between events increased from a median of 75 seconds to 98 seconds following CPA in the caudate putamen, the opposite effect on inter-event time as DPCPX. However, in the prefrontal cortex (Fig. 8C and 8D), CPA did not significantly change the underlying distribution and the inter-event times were not different (*n* = 6 rats, KS test, *p* = 0.9299). Thus, A_1_ activation with CPA decreased the frequency of spontaneous transient adenosine release in the caudate-putamen but not the prefrontal cortex.

## Discussion

Spontaneous, transient adenosine release occurs in both the caudate-putamen and prefrontal cortex. Transients were on average a couple hundred nM, which is sufficient to activate adenosine receptors [38]. Adenosine was elevated for only a few seconds and these are the most rapid adenosine changes that have been measured *in vivo*. While adenosine transients occurred on average once every several minutes, at least 30% of transients were less than one minute apart and there was no regularity to adenosine release. A_1_ receptors modulated the time between adenosine events but not the concentration. These studies reveal that large amounts of adenosine can be quickly released and cleared from the extracellular space, a contrast to previous studies which have documented a slower role for adenosine [39]–[41]. Thus, adenosine has a rapid signaling mode that may locally cause transient neuromodulation.

### The Concentration of Transient Release is Sufficient for Adenosine Receptor Activation

The average concentration of adenosine released was 180 nM, but the amount varied widely from 40 nM to 3.2 µM. Spontaneous adenosine release was the same order of magnitude as stimulated release in brain slices from the cerebellum [9], caudate-putamen [42] and prefrontal cortex [43]. However, evoked adenosine release in the caudate-putamen of anesthetized rats was typically 600–900 nM [11], [12], larger than the average spontaneous transient. Electrical stimulation likely activates all cells but different firing rates or number of cells activated during spontaneous adenosine release could lead to lower release.

In the prefrontal cortex, the concentration of adenosine release was significantly higher than in the caudate, although the difference was not large enough to suggest that different types of adenosine receptors are being activated in the two brain regions. Inhibitory A_1_ receptors and excitatory A_2a_ receptors both have affinities in the low nanomolar range [44] and have high to intermediate distributions in the caudate and prefrontal cortex [45]. About one percent of transients had concentrations greater than 1 µM, which could be sufficient to activate A_2b_ and A_3_ receptors [46]. These large transients demonstrate the potential for transient, micromolar adenosine signaling and high amounts of receptor activation.

### Spontaneous Adenosine Release is Random and the Frequency is Regulated by A_1_ Receptors

Adenosine release occurred on average once every 3–4 minutes; however, about half of the transients occurred within two minutes of each other in the caudate and one minute of each other in the prefrontal cortex. Spontaneous adenosine release is not periodic and consequently is unlikely to be caused by pacemaker firing or to directly modulate a rhythmic process such as breathing or tonic cell firing [47], [48]. Instead, release was random and the inter-event times fit an exponential decay, which arises from a discrete Poisson process [49] where each transient is not dependent on the prior adenosine event. The shortest time between transients was less than one second, indicating a long time is not required to reset adenosine release (Fig. 5A and 5B insets). Some of the larger transients may be two or more release events that occur simultaneously and cannot be temporally resolved. The frequency and concentration of release did not change over three hours, demonstrating that transient adenosine continues for long periods of time and that adenosine release is not just a response to immediate damage after electrode implantation [9], [13], [50].

A_1_ receptors are inhibitory adenosine receptors with low nM affinities that downregulate cAMP, hyperpolarize neurons, and can be neuroprotective during ischemia [38]. The cortex and striatum have intermediate to high levels of A_1_ receptor expression [45]. Previously, A_1_ receptors were shown to have autoreceptor characteristics and regulate the amount of stimulated adenosine release in the caudate *in vivo* [12]. Here, A_1_ receptors modulated the frequency of spontaneous adenosine release but not the concentration. DPCPX, an A_1_ antagonist, decreased the inter-event times in both brain regions. CPA, an A_1_ agonist, had the opposite effect and increased the inter-event times in the caudate-putamen but did not have a significant effect in the prefrontal cortex. A_1_ receptors are part of a feedback loop controlling transient adenosine release in the brain, where activation of A_1_ receptors decreases transient adenosine events. A_1_ receptor modulation does not affect concentration suggesting that the receptors do not control synthesis or the amount packaged into vesicles. Instead, A_1_ receptors modulate event frequency, such as regulating the number of docking or release events.

### Spontaneous Adenosine Release Occurs in Multiple Brain Regions

Spontaneous, transient adenosine release was measured in both the caudate-putamen and prefrontal cortex. Basal adenosine levels are higher in the prefrontal cortex than the caudate-putamen [51]–[53]. Similarly, the concentration of transient adenosine release is larger in the prefrontal cortex. However, transient adenosine release is unlikely to contribute to the basal levels of adenosine because of the rapid clearance. Spontaneous release was more frequent in the prefrontal cortex than in the caudate, which is similar to electrically-evoked release which was also more frequently observed in the prefrontal cortex [43]. The higher frequency of adenosine release in the prefrontal cortex could be due to either additional release sites or more release events per site.

The spontaneous adenosine transients measured here in the rat caudate and cortex were remarkably similar to the spontaneous adenosine transients measured in murine slices from lamina II of the spinal cord by Zylka’s group [13]. Despite the differences in brain region, preparation (slices vs. *in vivo*), and species, the average concentrations were the same order of magnitude (180 nM in rats compared to 570 nM in mouse slices). Differences could be a result of different numbers of cells activated or the amount of adenosine available for release. The average frequencies of spontaneous adenosine transients were also similar, with events occurring every two to three minutes. The comparable adenosine transients in different regions of rats and mice, along with previous electrophysiology work in the hippocampus implicating fast adenosine release regulating glutamate receptor excitability [7], show that transient adenosine release is a common feature in the nervous system. These transients could be an important mechanism of adenosine signaling, facilitating rapid neuromodulation throughout the brain.

### Spontaneous Adenosine Release Provides Local, Transient Modulation

Spontaneous adenosine signaling was fast, with the average transient lasting only about three seconds. This mode of transient signaling is a contrast to gradual adenosine buildup for minutes in the extracellular space during pathological events, such as ischemia [54]. Spontaneous adenosine transients were even more rapid than electrically-stimulated adenosine release, which elevated adenosine for up to 20 seconds in the caudate-putamen [11] or 100 seconds in the cerebellum [9]. Our time course matched well with previous measurement of transient adenosine release in spinal cord slices [13] and electrophysiology studies that observed a transient, 2 second variation in glutamate-evoked EPSPs after stimulated adenosine release [15]. The short duration demonstrates that the adenosine clearance mechanism from the extracellular space is very rapid, likely due to uptake [44]. Future studies will examine the effects of adenosine clearance mechanisms. Since the duration of adenosine release was the same in both brain regions, the mechanism of clearance is expected to be conserved between the caudate and prefrontal cortex.

The time course of spontaneous adenosine signaling is similar to that of transient, exocytotic release events of neurotransmitters. For example, dopamine signaling after phasic firing lasts only three to four seconds and dopamine is rapidly cleared from the extracellular space [55]. While our study did not address the pathway of extracellular adenosine formation, the mechanism is likely breakdown of exocytotically released ATP or direct, activity-dependent release of adenosine. Recent evidence shows transient, electrically-stimulated adenosine is activity dependent and a portion is not dependent on extracellular breakdown of ATP [9], [42]. Spontaneous adenosine release in slices of the spinal cord of mice is ATP-dependent [13], [14]. While the mechanism of spontaneous transient release is difficult to elucidate *in vivo*, future experiments could be performed in brain slices to determine if transient adenosine release is exocytotic or a downstream product of ATP.

The rapid release and clearance of adenosine has two consequences: adenosine can only signal locally and receptors will be activated transiently. The distance a molecule can travel in three seconds in the extracellular space is only 10–20 micrometers [56]. Therefore, only receptors close to the release event would be activated and transient adenosine release would provide local neuromodulation, close to the site of release. In addition, there could be heterogeneity within a region for receptor activation if not all areas experienced adenosine transients at the same time. The local variation of release was not directly assessed in these studies, but moving the electrode did change the number of adenosine transients detected suggesting a spatial effect on release events.

The function of rapid adenosine release is likely transient neuromodulation in the brain. Although most studies of adenosine function have not been performed on the seconds time scale, adenosine is known to regulate cerebral blood flow and neurotransmission on a longer time scale. It is likely that faster regulation of these processes is caused by transient adenosine release. Adenosine increases cerebral vasodilation in less than 60 seconds [57] and also increases cerebral blood flow [58]. Transient adenosine release could increase the flow of blood to localized areas in the brain that require an immediate boost in oxygen and nutrients. Adenosine regulation of neurotransmitter release can be inhibitory; for example, adenosine inhibits acetylcholine, glutamate, serotonin, dopamine, noradrenaline signaling by activation of A_1_ receptors [59]. Adenosine can also increase the release of acetylcholine and glutamate through A_2a_ receptor activation [59]. Although A_1_ and A_2a_ adenosine receptor affinities are in the low nanomolar range, the effective *in vivo* EC_50_ is estimated to be 600 nM [60], so changes on the order of hundreds of nanomolar concentrations could have significant physiological effects. This study shows that A_1_ receptors control the frequency of adenosine transients, but the downstream modulatory effects of the transient adenosine activation of A_1_ receptors are not known and should be explored in the future. A rapid, transient mode of adenosine signaling would provide discrete, local neuromodulation, which could facilitate fine control of neurotransmission.

## Conclusions

Spontaneous transient adenosine release was characterized for the first time *in vivo*. The spontaneous release in the caudate and prefrontal cortex is fast, lasting only a few seconds, and large, in the hundred nM range. The frequency of release was random, higher in the prefrontal cortex, and modulated by A_1_ receptors. These findings are a paradigm shift for understanding the time course of adenosine signaling. Previous studies have documented a long-term modulatory effect of adenosine during pathologies but here we demonstrate a new mode of transient adenosine signaling that could lead to rapid, local modulation. Future studies investigating the formation and function of this new type of adenosine signaling will reveal how adenosine modulates on the second time scale and regulates local brain function.
